# Inpatient Telemedicine Implementation as an Infection Control Response to COVID-19: Qualitative Process Evaluation Study

**DOI:** 10.2196/26452

**Published:** 2021-06-16

**Authors:** Nadia Safaeinili, Stacie Vilendrer, Emma Williamson, Zicheng Zhao, Cati Brown-Johnson, Steven M Asch, Lisa Shieh

**Affiliations:** 1 Department of Medicine School of Medicine Stanford University Stanford, CA United States; 2 Department of Engineering Stanford University Stanford, CA United States; 3 Department of Comparative Medicine School of Medicine Stanford University Stanford, CA United States; 4 Center for Innovation to Implementation Veterans Affairs Palo Alto, CA United States

**Keywords:** telemedicine, inpatient, COVID-19, qualitative, RE-AIM, infection control, personal protective equipment, implementation science, quality improvement

## Abstract

**Background:**

The COVID-19 pandemic created new challenges to delivering safe and effective health care while minimizing virus exposure among staff and patients without COVID-19. Health systems worldwide have moved quickly to implement telemedicine in diverse settings to reduce infection, but little is understood about how best to connect patients who are acutely ill with nearby clinical team members, even in the next room.

**Objective:**

To inform these efforts, this paper aims to provide an early example of inpatient telemedicine implementation and its perceived acceptability and effectiveness.

**Methods:**

Using purposive sampling, this study conducted 15 semistructured interviews with nurses (5/15, 33%), attending physicians (5/15, 33%), and resident physicians (5/15, 33%) on a single COVID-19 unit within Stanford Health Care to evaluate implementation outcomes and perceived effectiveness of inpatient telemedicine. Semistructured interview protocols and qualitative analysis were framed around the RE-AIM (reach, effectiveness, adoption, implementation, and maintenance) framework, and key themes were identified using a rapid analytic process and consensus approach.

**Results:**

All clinical team members reported wide reach of inpatient telemedicine, with some use for almost all patients with COVID-19. Inpatient telemedicine was perceived to be effective in reducing COVID-19 exposure and use of personal protective equipment (PPE) without significantly compromising quality of care. Physician workflows remained relatively stable, as most standard clinical activities were conducted via telemedicine following the initial intake examination, though resident physicians reported reduced educational opportunities given limited opportunities to conduct physical exams. Nurse workflows required significant adaptations to cover nonnursing duties, such as food delivery and facilitating technology connections for patients and physicians alike. Perceived patient impact included consistent care quality, with some considerations around privacy. Reported challenges included patient–clinical team communication and personal connection with the patient, perceptions of patient isolation, ongoing technical challenges, and certain aspects of the physical exam.

**Conclusions:**

Clinical team members reported inpatient telemedicine encounters to be acceptable and effective in reducing COVID-19 exposure and PPE use. Nurses adapted their workflows more than physicians in order to implement the new technology and bore a higher burden of in-person care and technical support. Recommendations for improved inpatient telemedicine use include information technology support and training, increased technical functionality, and remote access for the clinical team.

## Introduction

The COVID-19 pandemic created new challenges to delivering safe and effective health care while minimizing virus exposure among staff and patients without COVID-19. Telemedicine has long been recognized as a potential solution to these challenges and was deployed in response to the Ebola, SARS, and H1N1 outbreaks [1], and now the COVID-19 pandemic [2,3]. Health systems worldwide have moved quickly to implement telemedicine in diverse settings [4-8], drawing on existing best practices for implementing telemedicine to increase remote access [9]. Much less is understood about how best to connect patients who are acutely ill with nearby clinical team members, even in the next room. To guide this widespread deployment, a deeper understanding of inpatient telemedicine’s current use and potential impact on quality of care and team dynamics is needed.

Inpatient telemedicine adoption is also useful for personal protective equipment (PPE) conservation. For inpatients with known infections, the clinical team must don one-time-use PPE each time they enter the patients’ rooms to safely provide care and help prevent transmission. Increased demand for PPE has led to intermittent regional supply shortages that place millions of health workers at risk [10,11]. Inpatient telemedicine allows health workers to address patient needs without physically entering the room, thereby reducing exposure and preserving scarce PPE resources [2,12]. These consults occur on the same unit, making them qualitatively different from other telemedicine in that the clinical team is able to convert an initial virtual encounter to an in-person one should the need arise.

Health systems hoping to incorporate telemedicine must overcome several recognized barriers, including costs, training, resistance to change by clinical team members and staff, and patient characteristics associated with reduced comfort in using technology, such as older age or lower level of education [13,14]. The COVID-19 pandemic has helped facilitate the removal of some of these barriers, as the United States moved to relax regulations on interstate licensing and offered parity in reimbursement in many telemedicine settings [15]. Examples of this adoption by hospitalists to deliver inpatient care include system-wide deployment of an in-room video intercom system [16] and tablet-based web-conferencing systems [17]. Specialty care via telemedicine has also been explored in palliative medicine [18], urological care [19,20], and dermatology [21]. The United States is not alone, as telemedicine programs have been developed in other infection hot spot countries, such as China, Spain, and Italy, to minimize virus transmission [5,22,23].

Despite the growing implementation of inpatient telemedicine, formal evaluations are limited. Telemedicine used to deliver care remotely has been shown to be effective in many clinical settings [24]. In the more novel use of infection control, Borchert et al demonstrated that a portion of inpatient telemedicine urological consults were safely directed through a triage protocol that included a pathway exclusively for inpatient telemedicine care [20]. Given the nascent use of inpatient telemedicine, an exploratory understanding of how clinical teams are using inpatient telemedicine and the challenges they face is needed. Qualitative approaches are best suited for exploratory, ground-up understanding of emerging issues and situations within health services research [25]. In evaluating inpatient telemedicine, for example, qualitative methods have afforded us insights around how the technology is implemented and why it is or is not adopted. This paper is one of the first to seek frontline clinical team voices to understand how inpatient telemedicine is being implemented and its downstream effects on patient care, team dynamics, and perceived quality.

## Methods

### Overview

This study conducted 15 semistructured interviews with attending (5/15, 33%) and resident (5/15, 33%) physicians and nurses (5/15, 33%) on a designated COVID-19 unit at Stanford Health Care as part of a qualitative process evaluation. To clarify, attending physicians (eg, consultants) have completed all medical training and oversee resident physicians (eg, house officers) who have recently graduated from medical school and are in years 1 to 3 of their hospital training. This evaluation sought to understand the implementation and perceived effectiveness of an inpatient telemedicine solution across multiple domains, including patient care, clinical workflow, and clinical team satisfaction.

### Setting

Beginning in March 2020, a large academic health center—Stanford Health Care, Palo Alto, California, USA—designated a single inpatient nonintensive care unit to treat all potential and confirmed patients with COVID-19 admitted to their hospital. To reduce clinical team and staff pathogen exposure and to conserve PPE, each patient received an iPad tablet (Apple Inc) with the ability to receive web-conference calls from computers in two private conference rooms on the unit. A patient’s unique web-conferencing link and a password were required to enter the privacy-compliant system; this information was available to any clinical team members on the unit. Each tablet in the patient rooms had the web-based teleconferencing software Zoom (Zoom Video Communications, Inc) installed in a “hub-and-spoke” configuration, such that a call from the “hub” tablet on the unit floor would trigger the “spoke” patient tablet to automatically turn on its audio and video functions. The inpatient telemedicine intervention aimed to reduce clinical team in-person encounters and allowed the primary and specialist physician team, nurses, and extended care team members (ie, dietitians, respiratory therapists, physical therapists, social workers, and others) to provide patient care remotely in the on-unit conference rooms throughout the day.

### Instrument and Population

Semistructured interview protocols explored the implementation and perceived effectiveness of inpatient telemedicine in reducing pathogen exposure and PPE use. The interviews also captured perceptions of the appropriateness of inpatient telemedicine use in various settings and its impact on clinical team satisfaction. Interview protocols were developed using RE-AIM (reach, effectiveness, adoption, implementation, and maintenance), an implementation science framework most commonly used to support effective implementation of interventions, but that is also valuable in structuring the evaluation of key implementation outcomes [26-28]. Interview questions were open-ended and organized into themes around individual experience with the intervention, perceived patient and clinical team outcomes, changes to clinical team workflow, and anticipated future use cases of inpatient telemedicine beyond the COVID-19 pandemic. Interviews lasted approximately 30 minutes and took place by phone, except in the case of two interviews that were conducted in person in a private location on the COVID-19 unit. Though the team would have preferred to conduct all interviews in person, access to the unit was rightfully highly restricted requiring use of phone interviews.

The target population for interviews included nurses and resident and attending physicians who were part of the clinical team and had used telemedicine since its launch within the organization in March 2020. The evaluation team contacted the medical director and nursing manager on the COVID-19 unit and received a list of clinical team member names from which to contact potential participants and request an interview. Interviews were conducted between May and June 2020 and reflect technical capabilities and workflows used during this period. Purposive sampling methods were used to recruit participants until thematic saturation was reached. Recruitment was challenging due to the small pool of clinical team members working on the COVID-19 unit and their demanding responsibilities in the context of the pandemic. Saturation was reached, by role, when new themes no longer emerged during interviews [29].

### Analysis

Interviews were audio recorded and transcribed verbatim using Rev (Rev.com Inc). A qualitative health services researcher (NS) and a physician health services researcher (SV) independently reviewed and summarized key themes across all interview transcripts using a rapid analytic process structured around *a priori* categories informed by interview question categories (eg, structure, process, and patient outcomes) and RE-AIM constructs (Multimedia Appendix 1) [30]. Key themes and any inductive categories that emerged from this preliminary round of analysis were then validated using a consensus approach including an additional qualitative health services researcher on the evaluation team (CBJ) and two research assistants (EW and ZZ) who were also closely familiar with the data [31]. Each member of the team provided feedback on the others’ key themes, highlighted similarities and differences, and surfaced relevant participant quotes. Differences in data interpretation were reviewed and resolved only once a majority of the team came to consensus. The Stanford Institutional Review Board determined that this project did not qualify as human subjects research (Protocol 55927). As such, informed consent for participation was not required. However, each participant received a verbal description of the evaluation and was informed that their participation was voluntary and confidential.

## Results

### Overview

Using RE-AIM, as defined above, and categories drawn from the semistructured interview guide as an *a priori* coding framework for analysis, the following themes emerged: (1) implementation setting and climate, (2) clinical team workflows around inpatient telemedicine, (3) clinical team satisfaction with inpatient telemedicine, (4) perceived impacts on patients, (5) limitations of inpatient telemedicine, and (6) anticipated future uses of inpatient telemedicine. Table 1 describes the qualitative findings by theme and subtheme.

**Table 1:** Qualitative results by theme and subtheme and example quotes.

Theme and subtheme		Quote
**Implementation setting and climate**		
	Adoption	“Great. And who’s using this video chat option? Which types of roles are using it? Is it just attendings? Are residents involved? Nursing? Who else?” (Interviewer) “Yeah, I think everybody is, and I know nurses go in and out of the room too when I’m using it. So, everybody, essentially.” (Attending physician #2)
	Information sharing and training	“I believe I was told about it at a division meeting and I didn’t receive any formal training in it, but it was easy enough to use without having to go through a training session.” (Attending physician #3)
	Information sharing and training	“What information were you given when this all first launched about how to use the technology?” (Interviewer) “Not a lot. I was told it was available and that most patients with COVID had it as long as they were on the right units. And then that it was allowed in lieu of the physical exam now. So that was sort of the information that we were given.” (Resident physician #2)
**Clinical team workflows**		
	Physician workflow	“Typically what we do is as a team, we’ll go into one of the Zoom rooms and we will call the patient using the video chat. We’ll call in an interpreter if we need, and we’ll do the history taking and a visual physical exam over Zoom. And if the patient is new to me or had a clinical change that requires a physical exam, then after we do the rounding over the video chat, then I’ll go into the room and do any parts of the physical exam that require me to be physically in the room.” (Attending physician #4)
	Nurse workflow	“The organization is really depending on nurses to be solely, I guess the person who’s doing direct patient care most of the time in COVID rooms. Before this there were very few barriers to having physical therapy in the room or consulting teams doing their assessment and chatting with the patient, having housekeeping come in, right? But now we’ve taken over some of respiratory therapy’s responsibilities just to decrease exposure for them and to conserve PPE [personal protective equipment], so we’re doing metered-dose inhalers, et cetera, for them. Housekeeping isn’t allowed into the room except to do discharge. So we’re doing trash and linen for the moment. I mean, physicians will come in if it’s emergent and there are definitely different teams that come in to do their daily assessments, but for the most part it’s solely nursing doing physical assessments and they’re really relying on us to see the changes and advocate for the patients if something is new or if they’re deteriorating, et cetera.” (Nurse #5)
	Nurse workflow	“...in the beginning when the nurse comes in, before she goes in the room, she’ll do a teleconference with the patient to check on them, see if they’re awake, if they’re ready to order breakfast. What do they need before we come into the room, because we’re trying to compile care, kind of do as much as possible when we enter, so we’ll do it in the morning. We’ll bring them the breakfast tray, we’ll get them fresh linen, we’ll get them the morning medicine. So, we’re doing as much as possible when we go in the patient’s room.” (Nurse #3)
	Extended care team workflow	“...You have to first log in with the patient and then you say, ‘Hey.’ I speak Spanglish. I’m like, ‘Hola. Un minuto.’ And then I get the information for the chat, the number and the password, and I text it to the interpreter and then they hop in and some are between five and 10 minutes.” (Attending physician #2) “So in that five- to ten-minute gap, what happens?” (Interviewer) “Yeah, that’s awkward. And if you leave the room then the password changes. So, you have to stay there. I say in my Spanglish, ‘A person’s coming,’ and then we just kind of hang out. But actually, today the patient fell asleep, literally while we were sitting there because I think it took like nine minutes.” (Attending physician #2)
	Technology support workflow	“You mentioned a few things that weren’t going well already, but what else with the Zoom technology isn’t working well?” (Interviewer) “I think those were the main things. Like there were a couple times when it just like, was on pause or out of batteries or something...” (Resident physician #3) “Okay. And who’s actually responsible for maintaining the iPads? Like charging them?” (Interviewer) “I don’t know.” (Resident physician #3)
**Clinical team satisfaction**		
	Physician satisfaction	“For me, it helps the anxiety a lot. I have a young child at home, so related to that, I was really extra worried about becoming sick myself. Once I was actually in the hospital for the first time during COVID and seeing how smoothly things were going with the PPE and everything, I felt a little better already. But then just knowing that I didn’t need to be exposed any more than absolutely necessary. That was very helpful.” (Attending physician #5)
	Physician satisfaction	“So when I was on wards, I never went physically in the room with any of these [COVID-19] patients and in that way I think it’s detrimental...you don't get a lot of the teaching about the physical exam that you probably wanted.” (Resident physician #3)
	Nurse satisfaction	“It’s a little time consuming to be able to coordinate [calls]. [Physicians ask] ‘Oh, can you show me how to Zoom? Can you show me?’, even though we have clear instructions in each of those Zoom rooms, each of the conference rooms on how to set it up, everyone’s like, ‘Oh, can you show me how to do it?’ So, we have to kind of stop what we’re doing if it’s nothing too important and we have to go.” (Nurse #1)
**Perceived impacts on patients**		
	Quality of care	“I think from a like, is the patient getting the care that they need and are they getting better standpoint? The answer is yes, but I do think you lose something by not being able to be physically present next [to] your patient. And that might be something that’s like a, it’s like an intangible, but it’s kind of just like the, having the proximity of being a physician next to you to comfort you or reassure you, which is just different when you’re doing things remotely.” (Attending physician #3)
	Quality of care	“I think it puts a lot of onus on the nursing staff, which is okay because there is clinically a really strong staff, but I just wonder if that is the safest quality of care...That is why I don’t know if it really is good. I mean, yeah. I’m a little bit indifferent about it...I think in certain scenarios, it is really important and probably others, it is probably not. I do think there is potential for something to be missed. I worry, I guess.” (Nurse #4)
	Patient privacy considerations	“Sometimes we would try calling first to ask on the room phone and to ask if it was a good time. Another thing is [the tablet] was angled such that it was at their faces, not anywhere else, and so we felt like it was unlikely that they were going to be kind of like exposing that much of themselves. That was a challenge I don’t think I’ve fully solved.” (Attending physician #5)
	Patient privacy considerations	“I don’t think [privacy]’s an issue at all, and I could see how it’d be a question, but...to be honest with you, if you had to wait for the patient to answer, it just wouldn’t work. Most of the time they’re sleeping, even the middle of day, or they just don’t hear it...So if you needed them to answer the call, then it would only work 50 percent of the time.” (Attending physician #2)
**Limitations of inpatient telemedicine**		
	Communication	“[Researchers] were consenting [the patient] for remdesivir and they used an interpreter. They didn’t go into the room and they used an interpreter via phone and there was still a language barrier where the patient didn’t understand what they were consenting him on. So, he said no to remdesivir... I speak Spanish, so the next day I went into the room and I said, ‘Just a question. Why did you say no to the remdesivir trial?’ And he’s like, ‘I don’t know. I couldn’t really understand what they were saying over the phone, so I just said no.’” (Nurse #1)
	Patient–clinical team connection	“I had one COVID patient where I needed to have a goals of care conversation that really didn’t feel like it was working well over the Zoom, and so I did that in the room. I think I worry a little bit sometimes that if we only see patients over the Zoom, that it increases their sense of isolation and their feeling of fear and feeling... Yeah, their feelings of isolation and fear. And that there’s something to be said for the emotional connection and support that comes from physically being in the room.” (Attending physician #4)
**Anticipated future uses of inpatient telemedicine**		
	N/A^a^	“I do think that especially if it were something that we could access from all over the hospital...now that we have a new hospital and our patients are not well co-localized, people spend so much time just walking from place to place. I could see it really being helpful. Of course you want, under normal circumstances, to see every patient in person at least once a day, but if you’re literally a mile away in the hospital and a patient just has a question, it does feel more personal to Zoom in and be able to talk to them where they can see your face...Of course you get more information too, you can see, are they having trouble breathing? Do they look sicker than when you saw them earlier? That kind of thing.” (Attending physician #5)

^a^N/A: not applicable; there were no subthemes for this theme.

### Implementation Setting and Climate

Implementation setting and climate subthemes addressed adoption, information sharing, and training.

#### Adoption

Adoption of inpatient telemedicine was reported to be near universal across attending and resident physicians and nurses who cared for patients on the COVID-19 designated inpatient unit. Web-conferencing was also used to replace morning rounds for patients with COVID-19, which would otherwise take place with the full physician team (both residents and attendings) and nurses, when available.

#### Information Sharing and Training

Information sharing and training about the intervention were reported to be informal. The novelty of the inpatient telemedicine system also led to variations in the terminology used to describe it. Clinical team members called it “video visits,” “virtual rounding,” “inpatient telemedicine,” “telemedicine,” “video chat,” “Zoom visits,” or “Zoom.” There were no consistent terms used within interviewee roles, and no miscommunications resulting from variation in terminology were reported.

Most clinical team members learned about the inpatient telemedicine system through word of mouth, though some did participate in a technical training session, and instructions for initiating inpatient telemedicine visits were posted on the computers in the designated web-conferencing rooms. Most physicians reported learning about the technology from either the nursing team or from a physician coming off a previous shift. Most nurses also reported learning about inpatient telemedicine through their peers, though one had received training from an information technology (IT) representative. Though instructions were posted, nurses shared that they regularly received an influx of questions and requests for help initiating visits from physician team members and others. Nurses reported that these questions and requests did not seem to diminish in the months following implementation.

### Clinical Team Workflows Around Inpatient Telemedicine

#### Overview

Clinical team workflow subthemes addressed the relative stability of physician workflows compared to the period before the COVID-19 pandemic, the significant adaptations of nurse workflows to account for additional responsibilities associated with COVID-19 restrictions and inpatient telemedicine, workflows for extended care team members hinging on call coordination and availability of translators, and a need for improved technology support workflows.

#### Physician Workflow

Attending physicians and senior residents shared that they entered patient rooms less frequently due to the availability of inpatient telemedicine and, as a result, they reduced their PPE use and exposure to COVID-19. Junior residents did not enter the rooms at all per trainee program guidelines.

Clinical team members reported that a baseline physical exam was typically conducted in person by the senior resident or attending physician. Any additional in-room examinations were conducted by the nurse, senior resident, or attending and took place anywhere from daily to every few days, depending on the patient’s clinical needs. When asked how they decided whether or not to enter a room for an in-person assessment, physicians considered patient acuity of illness, opting to forgo low-value in-person encounters that could otherwise be addressed via video. Any decrement in clinical status also triggered an in-person exam. Rounding activities typically included daily telemedicine conversations between the patient and the physician team (ie, attendings and residents), as long as patients were clinically stable.

#### Nurse Workflow

Patients continued to receive regular, in-room nursing care multiple times each day. In addition to their usual responsibilities, nurses reported being tasked with additional responsibilities outside their standard work. These included delivering food multiple times per day, changing linens, and disinfecting patient rooms due to room entry restrictions barring housekeeping and cafeteria staff from entering the rooms of patients with COVID-19. Nurses reported “batching” their entrances to complete several tasks at once to reduce PPE use, and they used inpatient telemedicine when they wanted to check on patients but deemed an in-room encounter unnecessary. Nurses shared that inpatient telemedicine was primarily used for hourly assessments of each inpatient, which is considered best practice in nursing care, and was often a satisfactory alternate to an in-person encounter.

Most notably, nurses reported expending significant time to facilitate team use of inpatient telemedicine, including scheduling the virtual meetings and circulating web-conferencing links and passwords to all participants (eg, clinical team, interpreters, family members, and extended care team members). The coordination of a single call was reported to take up to 20 minutes of a nurse’s time to complete. While some attendings and residents reported setting up calls themselves, some corroborated their ongoing reliance on nursing support to facilitate calls.

Nurses also reported being asked to don PPE and enter patient rooms during inpatient telemedicine visits to turn the tablet screen or increase tablet volume to optimize the interaction with the physician or extended team member in the conference, as patients frequently moved the tablet or adjusted its settings between visits. These situations negated any reduction in PPE consumption that the inpatient telemedicine system may have facilitated and transferred potential exposure from the caller to the nurse. While nurses did not express dissatisfaction with these changes directly, some nurses shared concern that time spent facilitating telemedicine encounters for other team members pulled them away from their own patient care responsibilities.

Nurse-led adaptations of the technology that supported patient care included the following: (1) acquiring an extra tablet at the nursing station through IT to be able to monitor and provide 24/7 visualization of a delirious patient with dementia who was a fall risk and (2) facilitating communication between patients and family members, particularly in acute care situations, if they had time to coordinate the conference room and the family member and patient schedules.

#### Extended Care Team Workflow

On-site extended care team members, such as respiratory therapists, dietitians, psychiatrists, social workers, research coordinators, and other team members, were not physically permitted on the unit and could, therefore, not initiate inpatient telemedicine visits through on-site conference rooms. Given these limitations, nurses were also sometimes asked to conduct respiratory therapy activities or fulfill other clinical needs on behalf of their remote care team members.

Call coordination via inpatient telemedicine was reported to rely on nurses to coordinate and required each relevant team member to be available simultaneously in their own confidential setting for the telemedicine visit to take place effectively. Many patients with COVID-19 did not speak English as their first language, and it was particularly challenging to schedule remote interpreters on telemedicine visits. Interpreters worked virtually in multiple settings using multiple technical platforms, often producing a 5- to 10-minute delay while the physician and patient waited in the teleconference room.

#### Technology Support Workflow

The logistics of using and maintaining tablets in patient rooms created new workflows for the clinical team. Each tablet was mounted on a cart to the side of the patient bed and nurses mentioned that the placement of the tablet sometimes interfered in patient care, specifically in conducting physical exams or providing treatment that required access to that side of the bed. Additionally, tablet maintenance meant that updates automatically pushed by the technology companies or a dead battery could put a tablet out of commission, without reported backup machines available on the inpatient unit that could immediately address the issue.

### Clinical Team Satisfaction With Inpatient Telemedicine

Satisfaction subthemes highlighted high attending and resident physician satisfaction specific to reduced infection exposure and improved efficiency in daily workflows, resident physician concerns with reduced educational opportunities, and no stated change in satisfaction for nurses.

#### Physician Satisfaction

Most physicians perceived inpatient telemedicine to have a positive impact on their daily work and reported that the platform was intuitive to use, given their experience with the technology in nonclinical settings. Physicians also perceived having additional time to check in on patients given the reduction in time spent walking between patient rooms. These factors, in addition to reduced PPE use and in-room COVID-19 exposure, were reported to contribute to improved job satisfaction and reduce feelings of anxiety around infection among attending and resident physicians.

In-room physical exams prior to the pandemic typically included attending physicians and senior and junior residents; however, during the pandemic, attending physicians and senior residents primarily conducted in-room physical exams with patients with COVID-19 alone. Junior residents mentioned missing educational aspects of the physical exam due to this reduction in training at the bedside, though they appeared to accept the small trade-off on education to prioritize their safety and were still able to engage with patients through inpatient telemedicine.

#### Nursing Satisfaction

Nurses did not report a change in job satisfaction or anxiety, as they perceived that their in-room exposure stayed at similar, if not increased, levels following the implementation of inpatient telemedicine due to the increased housekeeping and care responsibilities described above. As fewer members of the primary physician team and extended care team were allowed to enter patient rooms, these additional tasks fell on the nursing team to complete.

### Perceived Impacts on Patients

Perceived patient impact subthemes included consistent quality of care, privacy considerations, and limitations of inpatient telemedicine, particularly with respect to communication, physical exams, and patient–clinical team connection.

#### Quality of Care

Overall, inpatient telemedicine was perceived to be effective in reducing COVID-19 exposure and PPE use without significantly compromising quality of care. Patient outcomes, specifically around quality of care, were perceived to be of similar quality to in-person visits. Physicians and nurses reported that inpatient telemedicine facilitated improved care by allowing the clinical team to “eyeball” a patient’s physical status remotely throughout the day without donning PPE. Additionally, all physicians reported that the inpatient telemedicine system allowed for higher-quality care than care delivered via phones available at the patient’s bedside, as physicians were able to assess patients’ physical statuses and capture facial expressions otherwise missed through audio alone.

#### Patient Privacy Considerations

Physicians and nurses felt that patient privacy was protected, as calls could only be made in the designated conferencing rooms through the secure system. Some clinical team members mentioned feeling concerned about the auto-activation of video in the patient’s room and would call the patient by phone just before initiating a web-conferencing visit to alert the patient. However, some physicians felt the auto-accept feature was critical to provide timely care if patients were unable to activate video by themselves.

The clinical team reported that patients sometimes turned the tablet screen away from themselves for privacy reasons and reduced the volume, especially at nighttime when the screen brightness and sound might interrupt their sleep. This allowed patients to rotate the screen toward themselves when they were ready for the visit, but it also meant that sometimes calls from the clinical team were missed, as the patient did not notice or could not hear the team initiating the call. It was at these points that a physician might ask a nurse to don PPE and enter the room to turn the tablet screen toward the patient and increase the audio volume.

### Limitations of Inpatient Telemedicine

Some residents acknowledged concern that the lack of physical presence may compromise care, particularly for vulnerable patients with an altered mental status and hearing challenges as well as for non-English speakers. At the same time, a resident also acknowledged that telemedicine opened up new care possibilities for a deaf patient who was admitted with COVID-19 and could only read lips. Whereas PPE would have covered the resident’s mouth during an in-person exam, telemedicine allowed a conversation to take place.

Additional use cases where inpatient telemedicine was thought to be inappropriate around prognosis included when sharing bad news or communicating complex information in another language. Several nurses reported concerns of less appropriate use by the extended care team, such as a psychiatric evaluation completed over telemedicine for a patient with known dementia or a physical therapy evaluation for a patient who had trouble standing. Other adaptations made to adjust to virtual patient care were also reported as less ideal, such as directing patients to identify complex medical instruments or asking nurses to conduct exams typically done by a resident. Some physicians and nurses also noted the loss of in-person connection, worrying that it may inadvertently lead to worse outcomes or at least reduce patient satisfaction. One nurse felt that this loss of interpersonal connection went against her training.

Loss of connection was reported to be felt by the patients as well. The clinical team reported that few patients had commented on their inpatient telemedicine experience, though those who had received feedback shared that some patients were confused as to why physicians and nurses were not entering the room as often. Some clinical team members perceived that inpatient telemedicine made their patients feel more isolated.

### Anticipated Future Uses of Inpatient Telemedicine

Clinical team members agreed that inpatient telemedicine might expand beyond use in patients with COVID-19 in future use cases. Contexts favorable to telemedicine use included those with patients who were comfortable with the technology and were clinically stable and mentally coherent. When asked what percentage of daily encounters they prefer to conduct via inpatient telemedicine in the future, clinical team members shared preferences ranging from 0% to 80%. Suggested uses included leveraging inpatient telemedicine to allow remote (ie, off-unit) access to patients throughout the day and to connect patients with remote visitors.

## Discussion

### Principal Findings

This is the first qualitative analysis of inpatient telemedicine used to improve infection control using established implementation science frameworks that the authors are aware of. Findings suggest that telemedicine used as an infection control tool in a nonintensive care inpatient setting was generally accepted and adopted among attending physicians, resident physicians, and nurses. Clinical team members agreed that quality of patient care remained largely unchanged, particularly given the ever-present option to convert a telemedicine assessment to an in-person exam if clinical deterioration was suspected. Recognized limitations included challenges around clinical team communication and personal connection with the patient, perceptions of patient isolation, ongoing technical challenges, and certain aspects of the physical exam. Such barriers have been previously reflected in the literature alongside concerns of technical costs and unfavorable reimbursement [13,32,33]. However, this work goes further to suggest that telemedicine *used in the context of an infectious disease* may be uniquely more favored by the clinical team than its past uses, which predominantly focused on connecting patients to remote providers. In the infectious disease setting, inpatient telemedicine provides both an additional layer of defense against an infectious pathogen and an opportunity to visually connect “face-to-face” (ie, without a mask) with vulnerable patients in need of care.

### Inpatient Telemedicine Improvements Underway

Investigations to augment key aspects of the physical exam and others with a “remote hospital system,” such as a connected stethoscope, are ongoing in the institution. In addition, the institution is implementing solutions to the challenges around the patient–clinical team connection; ease of communication, particularly with the timeliness of incorporating interpreters, is in the process of being improved, following rapid feedback of these findings. These findings also point to the possibility of incorporating a calendar system for patients who are hospitalized who may see their schedules filled with procedures, specialist consults, and family conference calls. A series of remote but human connections throughout the day may ease reported feelings of patient isolation and simplify challenges related to multi-party scheduling.

### Variance in Level of COVID-19 Exposure, by Role

While stakeholders perceived that inpatient telemedicine is effective in preventing COVID-19 exposure and reducing PPE use for most clinical team members, perceptions varied by role. Physicians, especially junior residents, reported having the lowest degree of in-room exposure, as attendings and senior residents conducted the majority of in-person assessments. In-room exposure was reportedly highest among the bedside nursing team who spent more time in the room, as their responsibilities shifted to include ancillary duties (eg, food delivery, linen changes, and respiratory care) that would have otherwise been conducted by the extended care team; their experience is consistent with those in other health systems and countries that have highlighted the critical roles nurses have played in the COVID-19 response [34]. The overall impact that telemedicine availability had on the number of times nurses entered a room was not clear from interviews: while nurses adapted to increased responsibilities, they also reported “batching” these activities to reduce PPE use. This shift in behavior is consistent with previously documented nursing responses in infection settings [35]. Overall, telemedicine seemed to magnify pre-existing discrepancies of time spent at the bedside between roles [36], with physicians spending less time at the bedside and nurses spending the same or more time at the bedside.

Literature suggests this major shift in clinical workflow may not be without cost. Nursing interruptions are dangerous and have been shown to compromise patient safety and quality of care, particularly around wrong medication or wrong dose [37-39]. In this academic setting, early learnings have facilitated conversations with operational, IT, and nursing leadership, and solutions to minimize nursing interruptions are actively being investigated. Any such solution must also consider nursing goals to optimize patient care and experience, as literature suggests that nurses may prefer to oversee who enters the patient room, even if virtually, to avoid disrupting the patient’s sleep or to optimize coordinated communication with family [40-42].

### Need for Rapid Evaluation of Information Technology

Many have pointed out that for health care institutions to become learning health care systems, they must pair rapid innovation with rapid evaluation [43]. Perhaps this is nowhere more important than in the adaptation of IT during a pandemic. Stanford Health Care had previously developed a system for such rapid evaluation [44] and deployed it in real time as the system struggled to adapt to the strain of the influx of new COVID-19 cases. This allowed clinical managers to draw lessons quickly and fine-tune the socio-technological system to maximize patient quality and minimize infection risk. Other health care systems might employ similar techniques in implementing IT solutions.

These interviews with clinical stakeholders point to a set of key opportunities to improve inpatient telemedicine, including clinical team education, IT support and training during the transition phase, technical functionality around audio volume and video privacy, and remote access for staff. Recommendations for improved inpatient telemedicine use are outlined in Table 2.

**Table 2:** Recommendations for improved inpatient telemedicine use.

Opportunity for improvement	Recommendations
Information technology (IT) support and training	Robust and locally available IT support staff to manage tablet maintenance, including charging and updating software, and to support the education of key end users Technological onboarding for the extended care team should go beyond written instructions and include videos and real-time support
Increased technical functionality	Camera and microphone functionalities in the patient room may be further optimized by relocating devices to the ceiling or wall, out of the way of bedside care Remote direction change, zoom, and volume functionalities may reduce the currently reported need for nursing staff to enter the room to reposition and change tablet settings Additional functionality focused on patient privacy may include a camera that clearly points away from the patient when it is not in use and incorporates a “knock knock” feature prior to automatic answer
Remote access for staff	Remote inpatient telemedicine can increase patient-team connections and leverage a partially remote workforce by enabling the clinical team to check on patients from remote settings outside of the COVID-19 designated unit National policies surrounding parity in reimbursement for telemedicine services and privacy laws that are still under debate in the United States will determine the feasibility of this recommendation

### Limitations

This study was conducted within a single academic medical center undergoing operational changes in response to the COVID-19 pandemic, and learnings are, therefore, not generalizable to other institutions or time periods. The use of qualitative methods provided a more nuanced assessment of facilitators and barriers to inpatient telemedicine implementation and adoption; however, this study was limited by a small sample size representing each role, as the hospital appropriately restricted nonclinical staff on the COVID-19 unit of interest. While the evaluation team felt that thematic saturation for each population was attained, unexpected viewpoints from a broader population may have been missed. Additionally, though efforts were made to include patient voices in this evaluation, the authors were unable to complete a sufficient number of patient interviews to draw meaningful conclusions from this perspective. An exploration of the patient perspective and a quantitative analysis of inpatient telemedicine’s impact on patient outcomes are important areas of future work.

### Conclusions

In this evaluation of an inpatient telemedicine system deployed as an infection control measure, attending and resident physicians and nurses reported virtual encounters as acceptable and effective in reducing COVID-19 exposure and PPE use for certain clinical team members. Additionally, the clinical team perceived quality of patient care to remain unchanged, though challenges were identified around the following: increased burden of technical implementation borne disproportionately by nurses, technology support, integration of remote extended care team members, patient–clinical team communication and connection, and conducting physical exams. Ongoing optimization of the technical and team workflow aspects of inpatient telemedicine is needed to deliver safe, effective care during the current and future pandemics.

## Appendix

Multimedia Appendix 1 Semistructured interview protocol.

## Abbreviations

IT: information technology
PPE: personal protective equipment
RE-AIM: reach, effectiveness, adoption, implementation, and maintenance
