# Supplementary material for: Inpatient Telemedicine Implementation as an Infection Control Response to COVID-19: Qualitative Process Evaluation Study
Source: JMIR Form Res. 2021 Jun 16;5(6):e26452. doi: 10.2196/26452 (PMC8211098; doi:10.2196/26452)
Supplement: Multimedia Appendix 1 [file formative_v5i6e26452_app1.pdf]

Perspectives of the clinical team on inpatient telemedicine as an infection control response to COVID-19: A qualitative process evaluation

Supplemental Files: Semi-structured Interview Protocol

Section	Main question	Prompts	Link to concepts
Introduction	What is your role on this unit?	1. Which units do you typically round in?<br>2. What do you like best about your job?	Positive inquiry; Reach
Structure	We are interested in the inpatient telemedicine technology. What do you know about this?	1. Are there other names for this technology? (Telerounding, video visits, virtual rounding.)	Implementation
Process	How has the inpatient telemedicine* technology been incorporated into your workflow?<br><br>*Can use their own words to describe technology.	1. How do residents use inpatient telemedicine?<br>2. How do attendings use inpatient telemedicine?<br>3. How do nurses use inpatient telemedicine?<br>4. Who else uses the inpatient telemedicine? How does this work? (Physical therapists, specialist consultants, caregivers)	Implementation
	How do you decide when to use inpatient telemedicine?	1. When is it necessary to enter a patient's room who has a highly communicable disease? Who typically does this?<br>2. Are there some patients for whom you only use telemedicine? Why?	Implementation
	How is inpatient telemedicine going for you?	3. What about inpatient telemedicine is working well? How easy is it to use?<br>4. What are you able to do via inpatient telemedicine that you otherwise would not be able to?<br>5. What about inpatient telemedicine is not working well?<br>6. What would you change to improve these issues?<br>7. What are you unable to do during inpatient telemedicine that you would normally do during an in-person rounding visit?	Implementation; Effectiveness

	What does your team do to protect patient privacy when using the inpatient telemedicine technology ?		Implementation
Patient outcomes	How has inpatient telemedicine impacted patient care?	1. How does inpatient telemedicine enhance patient care?<br>2. How does inpatient telemedicine detract from patient care?	Effectiveness
	How have patients responded to inpatient telemedicine?<br>What about caregivers?	1. What feedback have you received from these groups about inpatient telemedicine?<br>How has it changed your communication with these groups?	Effectiveness
Provider outcomes	How has it impacted your job satisfaction?		Effectiveness
Future use cases	How should we use telemedicine going forward?	1. Which types of patients may benefit most?<br>2. Which types of health workers may benefit most?<br>3. In an ideal world, what proportion of all interactions with patients in your day should be through telemedicine?	Maintenance
